# Evaluation of Performance of Introduced Yam Bean (*Pachyrhizus spp.*) in Three Agro-Ecological Zones of Rwanda

**DOI:** 10.1007/s12042-017-9188-5

**Published:** 2017-05-25

**Authors:** Ndirigwe Jean, Rubaihayo Patrick, Tukamuhabwa Phenihas, Agaba Rolland, Rukundo Placide, Mwanga O. M. Robert, Tumwegamire Silver, Kamarirwa Vestine, Kayinamura Evrard, Wolfgang J. Grüneberg

**Affiliations:** 1 Department of Agricultural Production, College of Agricultural and Environmental Sciences, Makerere University, P.O. Box 7062, Kampala, Uganda; 2 Rwanda Agriculture Board, Roots and Tubers Programme, P.O. Box 7231, Kigali, Rwanda; 3 International Potato Center (CIP), P.O.Box 22274, Kampala, Uganda; 4 IITA Station, Dar-es-Salaam, Tanzania; 5 National University of Rwanda, P.O.Box 138, Huye, Rwanda; 6 International Potato Center (CIP), La Molina 1895, Apartado 155812, Lima, Peru

**Keywords:** *Pachyrizhus*, GxE, High storage roots yield, Harvest index, GGE bi-plot, Rwanda

## Abstract

The yam bean (*Pachyrizhus spp*) was recently introduced as a root crop with high-yield potential, considerable protein and micro-nutrient concentration to investigate its potential for food production in Rwanda. Except for Chuin types (*Pachyrizhus tuberosus*) which have high storage root dry matter (RDM) (26 to 36%), most accessions are consumed raw and are reported to have low RDM. The present study aimed to evaluate and identify adapted high yielding yam bean accessions in major agro-ecological zones of Rwanda. Field experiments with 22 accessions were conducted in 2012 at three research sites representing the major agro-ecologies of Rwanda. Strict reproductive pruning was followed to enhance fresh storage root yields. Across locations, ANOVA indicated highly significant differences (*p* < 0.01) for genotypes (G), locations (L), seasons (S) and G x L effects for storage root yield, vine yield and harvest index and accounted for 21.88%, 43.41%, 1.43% and 13.25% of the treatment sum of squares, respectively. The GGE bi-plot revealed that EC209018 is high yielding but unstable. However, genotypes, AC209034, AC209035 and EC209046, were outstanding in terms of adaptation and relative stability across the 3 locations, suggesting consistent root yields irrespective of location and environmental conditions. The GGE scatter plot showed that all genotypes formed one mega-environment for storage root yield (Karama, Musanze and Rubona) and two mega-environments for biomass yield (Karama and Rubona as one mega-environment and Musanze the second one). This study revealed that Karama is the most suitable environment for evaluation and selection of yam bean for yield components in Rwanda.

## Introduction

Yam bean (*Pachyrhizus spp.*,) is a small genus of storage root forming legumes with neo-tropical origin. The genus *Pachyrhizus* belongs to the subtribe *Glycininae* (Lackey 1977) and the closest major crop is soybean (*Glycine max*). The genus consists of five species (Sørensen 1990; Doyle and Doyle 1993) and three of these species are cultivated including; the Andean yam bean (*P. ahipa*), Mexican yam bean (*P. erosus*), and Amazonian yam bean (*P. tuberosus*) (Sørensen 1996). The three cultivated yam bean species can be easily intercrossed (2n = 22) resulting in fertile interspecific hybrids (Grum 1994; Grüneberg et al. 2003). Whereas other legume crops such as soybean and common bean (*Phaseolus vulgaris*) are grown mainly for their edible seeds, yam bean is grown mainly for its edible storage roots (Sørensen et al. 1997). The yam bean also produces seeds but are not edible because they contain high amounts of toxic polyphenols especially rotenoids. However, the extract of rotenoids has potential for use in insecticide products to control thrips, aphids and whitefly larvae in crop production (Lautié et al. 2013; Noman et al. 2007; Alavez-Solano et al. 1996).

In many countries of the Americas and Asia, yam bean is produced on small to medium scale and consumed as vegetable or fruit root (Grüneberg et al. 2003; Karuniawan 2004), whereas in Africa the crop is unknow on-farm. The cultivated yam beans produce heavy storage roots (10–15 cm diameter and up to 20 kg weight) compared to other root crops such as cassava (*Manihot esculenta*) and sweetpotato (*Ipomea batatas*). Storage roots contain high protein content (12.7 mg/100 g of dry matter), which is three to five times higher compared to traditional root crops such as Irish potato (2.0 mg/100 g) and cassava (1.36 mg/100 g) (Santayana et al. 2014; Montagnac et al. 2009; Zum Felde et al. 2009). The nutrient rich and nitrogen fixing yam bean has been recently introduced to Rwanda and other Central African countries to obtain information if the crop is adapted to this sub-region of the word. Yam bean is propagated by seeds and is self-fertilizing such that superior homozygous genotypes can be fixed and maintained by smallholders without buying new seeds (Grum 1994).

The yam bean storage roots are largely consumed as a raw vegetable salad. However, a new yam bean type (Chuin) from Peru belonging to *P*.*tuberosus has* high root dry matter content (Sørensen et al. 1997) and can be consumed like cassava (Grüneberg et al. 1998). Similar to cassava, yam bean accessions have been processed into “gari”, a flour product consumed by millions of people on a daily in West Africa (Zanklan et al. 2007). The yam bean flour has been found to have extreme high iron concentrations (3.4 mg/100 g) and can be used up to 40% in different wheat flour-based food products (Zanklan et al. 2007; Wassens 2011; Padonou et al. 2013; Adegbola et al. 2015). Yam bean is attractive to agronomists and plant breeders due to its potential of providing high and stable yields, propagation by seeds and ability to sustain many cropping systems due to its high capacity in enrichment of soils (Castellanos et al. 1997; Annerose 1998; Zanklan et al. 2007; Nusifera and Karuniawan 2007; Rodriguez-Navarro et al. 2009).

Yam bean has been very successfully introduced to various Asia countries as well as China (Sørensen 1996) and is considered as a well-established crop, for example, in Indonesia; the crop is grown on over 5000 ha which constitutes about 5% of the sweet potato cultivation. The yam bean was also introduced to various tropical and subtropical regions of West Africa to supplement food sources and enhance sustainability of farming systems (Belford et al. 2001; Zanklan et al. 2007; Padonou et al. 2013; Adegbola et al. 2015).

Across the Rwandan agro-ecological zones, agricultural productivity is constrained by low soil fertility characterized by limited soil potassium, phosphorus and nitrogen levels (Gibson and Aritua 2002; Gaidashova et al. 2009). Provided the yam beans are adapted in Rwanda, the crop could enhance sustainability of farming systems; improve crop production and soil rehabilitation. The acquisition of yam bean seed by several countries of East and Central Africa (i.e. Uganda, Rwanda, Burundi, and D.R. Congo) was made possible by the International Potato Center (CIP) to explore its potential for food production, processing and genetic improvement in this region of the world (Heider et al. 2011). The use of yam bean might alleviate the food insecurity and decrease the high malnutrition observed in children between 0 and 5 year (IFPRI 2007; WFP 2009; FAO 2012), which is most attributed to the over dependence on the major root and tuber crops [potato, cassava and sweetpotato] that are poor sources of absorbable micronutrients and proteins. Since introduction in 2010, the yam bean into Rwanda’s major agro-ecological zones, this study reports the first set of results on the potential for adaptation and utilization of yam bean storage roots in Rwanda. This paper focused on the results of storage root yield, genetic variability for yield and yield components, as well as GxE interactions for yam bean accessions introduced to Rwanda.

## Results

### Analysis of Variance

The main effect due to genotypes (G) was significant for SRFY, VNY, BIOM, and RDM, whereas the number of roots per plant did not vary among genotypes (Tables 1 and 2). The main effects due to locations were significant for all traits but seasons (S) effects were significant for number of roots per plant, SRFY, VNY, BIOM, number of roots and HI. The interaction effects of genotypes by locations by seasons (GxSxL), and genotypes by seasons (GxS) on number of storage roots, storage root yield (SRFY) and vine yield (VNY), total biomass (BIOM), harvest index (HI) and storage root dry matter (RDM) content were not significant (Table 2). The genotypes by location interactions (GxL) were significant for SRFY, VNY, BIOM, HI and RDM. The location by season (LxS) was significant for number of roots and VNY.

**Table 1:** Description of yam bean accessions used in the study

Accession No	Accession code	Species	Plant type	Origin
1	AC209003	P.*ahipa*	Bushy-erect	Bolivia
2	AC209004	P.*ahipa*	Bushy-erect	Bolivia
3	AC209006	P.*ahipa*	Bushy-erect	Mexico
4	AC209007	P.*ahipa*	Bushy-erect	Guatemala
5	EC209016	*P. erosus*	Climbing	Mexico
6	EC209017	P. erosus	Climbing	Mexico
7	EC209018	P. erosus	Climbing	Mexico
8	EC209019	P. erosus	Climbing	Mexico
9	AC209022	P.*ahipa*	Bushy-erect	Bolivia
10	AC209023	P.*ahipa*	Bushy-erect	Bolivia
11	AC209024	P.*ahipa*	Bushy-erect	Bolivia
12	AC209031	P.*ahipa*	Bushy-erect	Bolivia
13	AC209032	P.*ahipa*	Bushy-erect	Bolivia
14	AC209033	P.*ahipa*	Bushy-erect	Bolivia
15	AC209034	P.*ahipa*	Bushy-erect	Argentina
16	AC209035	P.*ahipa*	Climbing	Mexico
17	EC209036	P. erosus	Climbing	Mexico
18	EC209046	P. erosus	Climbing	Mexico
19	EC209050	P. erosus	Climbing	Mexico
20	EC209052	P. erosus	Climbing	Mexico
21	TC209054	*P. tuberosus*	Climbing	Brazil
22	TC209060	P.tuberosus	Climbing	Brazil

AC: Andean yam bean (*P. ahipa*), EC: Mexican yam bean (P*. erosus*) and TC: Amazonian yam bean (P.*tuberosus*)

**Table 2:** Mean square estimates by ANOVA for number of storage roots, yield of storage roots (SFRY) and vines (VNY), total biomass, harvest index (HI) and dry matter content of storage roots (DMC) form a series of experiments over three locations and two seasons with yam bean under Rwandan growing conditions

Source	DF	Number of roots	SFRY (t/ha)	VNY (t/ha)	Biomass (t/ha)	HI (%)	DMC (%)
G	21	115.8	5999.7**	2370.3*	15,745.6**	5164.4	241.6*
L	2	297.5**	11,903.4**	5270.2**	32,867.8**	4164.6*	1033.2*
S	1	73.7*	393.5**	408.1**	1603.1**	933.2*	189.3
SL	2	14.27*	22.7	0.68	22.3	111.1	234.7
R:SL	12	46.5*	426.5*	417.4**	1552.4**	2811.7	363.3
GS	21	15.8	490.1**	220.9*	1317.1**	909.5	240.9
GL	42	75.0	3634.9**	2583.2**	11,674.1**	17,830.2**	141.0*
GSL	42	8.0	367.5**	240.6	1013.2	6463.7	89.6**
RGSL	249	20.9	4177.6	3214.0	12,898.5	43,630.7	1146.0

The degree of freedom for residual is 249 and not 252 due to three missing data

*DF* degrees of freedom, *G* Genotypes, *L* Locations, *S* Seasons, *SL* Seasons by Locations, *R:SL* block of replications within Seasons and locations, *GS* genotype by season interaction, *GL* genotype by location interaction, *G S L* genotype by season by location interaction and *RGSL* Residual of Genotypes (G), seasons (S) and locations (L) effects

*Significant at the 0.05 level

**Significant at the 0.01 level

Across locations and seasons, the genotype mean estimates for number of storage roots, SRFY, VNY, BIOM, HI and RDM are shown in Table 3. The mean number of storage roots varied between 1.0 (AC209035) and 1.7 (for AC209006). Accessions with high mean number of storage roots were AC209004 (1.5), AC209023 (1.4) and AC209024 (1.4). The average SRFY were highest for accessions EC209018 (25.5 t ha^−1^) and AC209033 (17.1 t ha^−1^) and lowest for accessions, EC209052 (7.8 t ha^−1^) and TC209054 (6.5 t ha^−1^). Also, VNY means were highest for accessions EC209018 (15.9 t ha^−1^) and AC209033 (12.0 t ha^−1^) and lowest for accession TC209054 (4.8 t ha^−1^). The yield advantage of EC209018 over all accessions corresponds to superior HI (65.8) (Table 3). BIOM means were high for accessions EC209018 (41.4 t.ha^−1^), AC209033 (29.1 t ha^−1^), AC209035 (28.4 t.ha^−1^), AC209034 (23.5 t ha^−1^) and AC209032 (22.6 t ha^−1^) and lowest for accession TC209054 (11.3 t.ha^−1^). For all tested accessions HI higher than 50% were observed, with the highest HI mean estimates for accessions EC209018 (65.8) and AC209022 (64.2%). The highest RDM of 26.4% and 25.0% were observed for accessions of TC209054 and TC209060, respectively, while the lowest RDM was found in accession AC209033 (14.4%).

**Table 3:** Accession mean estimates for number of storage roots, yield of storage roots (SFRY) and vines (VNY), total biomass, harvest index (HI) and dry matter content of storage roots (DMC) form a series of experiments over three locations and two seasons with yam bean under Rwandan growing conditions

Accessions	Number of roots	SFRY (t ha^−1^)	VNY (t ha^−1^)	Biomass (t ha^−1^)	HI (%)	DMC of storage roots (%)
AC209003	1.3	9.8	7.6	17.4	50.3	16.7
AC209004	1.5	8.5	6.0	14.5	56.1	17.0
AC209006	1.7	12.5	9.4	21.9	59.0	17.5
AC209007	1.4	10.0	7.7	17.7	54.8	22.3
EC209016	1.1	10.4	6.5	16.9	63.3	20.8
EC209017	1.1	11.7	8.1	19.8	59.1	18.8
EC209018	1.0	25.5	15.9	41.4	65.8	17.9
EC209019	1.1	10.7	8.3	19.1	57.3	19.2
AC209022	1.1	11.3	7.4	18.7	64.2	19.1
AC209023	1.4	13.2	8.8	22.0	58.8	16.2
AC209024	1.3	9.4	6.2	15.6	61.5	21.6
AC209031	1.1	12.6	8.7	21.3	58.5	17.3
AC209032	1.1	13.6	9.1	22.6	62.9	17.5
AC209033	1.1	17.1	12.0	29.1	58.5	14.4
AC209034	1.1	13.4	10.2	23.5	61.6	16.5
AC209035	1.0	16.6	11.8	28.4	59.3	15.6
EC209036	1.1	9.0	6.5	15.6	57.1	18.5
EC209046	1.1	11.7	8.5	20.3	59.4	18.2
EC209050	1.3	12.2	8.2	20.4	62.6	22.5
EC209052	1.1	8.0	5.5	13.4	55.8	21.5
TC209054	1.2	6.5	4.8	11.3	54.0	26.4
TC209060	1.3	9.0	6.1	15.1	57.3	25.0
Mean	1.2	12.0	8.3	20.3	59.0	19.1
LSD (0.05)	0.1	3.4	2.3	5.5	4.5	1.1

### Variance Components and Heritability of Yield and Yield Components

For all traits, the variance component due to genotypes (σ_G_^2^) was different from zero (Table 4). The σ_G_^2^ were highly significant (*P* < 0.01) for SRFY, BIOM, and DMC, significant for VNY (*P* < 0.05), but non-significant for HI and number of roots. The variance components σ_L_^2^ and σ_S_^2^ were highly significant for SRFY, VNY, and BIOM. The σ_L_^2^ for DMC was significant while genotype by location interactions (σ_G×L_^2^) were highly significant (*P* < 0.01) for SRFY, VNY, BIOM and HI, significant for DMC and not significant for number of storage roots per plant. The three-way interaction (σ_G×S×L_^2^) variance component was only significant for SRFY and DMC. Large σ_L_^2^ were observed for BIOM (88.0) and SRFY (31.9). The ratio of σ_G×L_^2^/σ_G_^2^ was larger than one for HI and VNY, whereas this ratio was small than one for BIOM, SRFY, RDM, and number of roots. The operational broad-sense heritability (*h* ^*2*^) for number of roots, YLD, VNY, BIOM, HI and DMC were 0.28, 0.64, 0.41, 0.57, 0.01 and 0.90, respectively.

**Table 4:** Variance component estimates and operational broad-sense heritability for number of storage roots, yield of storage roots (SFRY) and vines (VNY), total biomass, harvest index (HI) and dry matter content of storage roots (DMC) form a series of experiments over three locations and two seasons with yam bean under Rwandan growing conditions

Trait	σ_G_^2^	σ_L_^2^	σ_S_^2^	σ_L×S_^2^	σ_G×L_^2^	σ_G×L_^2^/σ_G_^2^	σ_G×S_^2^	σ_G×S×L_^2^	σ_ε_^2^	*h* ^2^
Number of roots	4.2	3.7**	0.1*	0.14	1.4	0.4	0.00	0.1	1.6	0.28
Storage root yield	4.7**	31.9**	1.6**	0.2	3.1**	0.7	2.7**	0.8**	1.5	0.64
Vines yield	1.9*	14.1**	1.7**	0.2	2.2**	1.2	0.4*	0.6	1.2	0.41
Biomass	12.3**	88.0**	6.6**	0.8	9.9**	0.8	2.1**	0.5	4.6	0.57
Harvest index	4.0	11.1*	3.8*	1.6	16.1**	4.0	3.9	12.1	15.6	0.00
DMC of storage roots	4.8*	5.1*	0.1	0.2	2.3*	0.5	0.2	2.0**	3.2	0.90

σ_G_^2^: variance components of genotypes, σ_L_^2^: variance component of locations, σ_S_^2^: variance components of season, σ_L× S_^2^: variance components of location by season interaction, σ_G×L_^2^: variance component of genotype by location interaction, σ_G× S_^2^: variance component of genotype by season interaction, σ_G×S×L_^2^: Variance components of interaction of genotype, season and location, σ_ε_^2^: error; *h* ^2^: operational broad-sense heritability

*Significant at the 0.05 level

**Significant at the 0.01 level

### Adaptability and Yield Stability of Tested Yam Bean Accessions across Six Environments

The subdivision of G × E sum of squares (Table 5) into heterogeneity of regression and deviations from regression analysis for traits that exhibited considerably larger σ^2^_G×E_ than σ^2^_G_ or σ_ε_^2^ revealed highly significant (*P* < 0.01) variance (σ^2^) components for SRFY with respect genotypes and environments, for BIOM with respect to genotypes, and for HI (*P* < 0.05) with respect to environments. Number of roots per plant, SRFY, VNY, BIOM and DMC were observed to be high at Karama across both seasons (Table 6), while for Musanze low means were observed for number of roots per plant, SRFY, VNY, BIOM, HI and DMC. The largest HI (65.5%) was observed at Rubona. SRFY means were generally higher in season B (19.0 t. ha^−1^) than season A (4.0 t. ha^−1^). The overall mean for number of storage roots, SRFY and VNY, BIOM, HI and DMC of yam bean across sites were 1.0, 12.0 t.ha^−1^, 8.3 t.ha^−1^, 20.3 t.ha^−1^, 59.0% and 17.4%, respectively.

**Table 5:** ANOVA for genotype (G) by environment (E) interaction (G × E) with subdivision (SUB) of G × E interactions using regression analysis for storage root yield, harvest index and biomass

Trait	Effect	df	MS	σ^2^	†Rel. σ^2^
Storage root	E	5	2463.9	36.4**	270
Yield	G	21	285.7	13.5**	100
	G × E	105	42.8	8.7**	64
	SUB × Hert. R. × G	21	132.5	6.2**	72
	Dev. R. × G	84	20.4	1.2	14
	Hert. R. × E	5	518.1	7.6**	87
	Dev. R. × E	100	19.0	0.7	9
	Error	249	16.8	16.8	124
Harvest index	E	5	1 033.2	11.1*	37,000
	G	21	241.6	0.03	100
	G × E	105	240.9	21.9*	73,000
	SUB × Hert. R. × G	21	363.3	8.5*	39
	Dev. R. × G	84	210.4	11.7	53
	Hert. R. × E	5	2466.4	35.4**	162
	Dev. R. × E	100	129.7	−15.2	−15
	Error	249	175.2	15.6	52,130
Biomass	E	5	6898.7	101.3**	34
	G	21	749.8	34.2**	100
	G × E	105	133.4	27.2**	79
	SUB × Hert. R. × G	21	407.4	19.0**	70
	Dev. R. × G	84	64.9	4.4*	16
	Hert. R. × E	5	1687.2	24.7**	91
	Dev. R. × E	100	55.7	1.3	5
	Error	249	51.8	51.8	151

The degree of freedom for error has to be 252 but it is 249 due to three missing data

*Hert. R.* heterogeneity due to regression, *Dev. R.* deviations from regression lines, *Rel. σ* ^*2*^ *Rel. σ* ^*2*^ Relative to σ_G×E_^2^

*Significant at the 0.05 level

**Significant at the 0.01 level

**Table 6:** Mean of number of storage roots, yield of storage roots and vines, total biomass, harvest index and dry matter content of storage root of yam bean in each environment

Traits	Environment						Means
	Rub.A	Rub.B	Kar.A	Kar.B	Mus.A	Mus.B	
Number of roots	1.1	1.3	1.5	1.3	0.4	0.5	1.0
Storage root yield (T. ha^−1^)	11.3	14.0	17.5	19.0	4.0	5.8	12.0
Vine yield (T. ha^−1^)	7.1	9.1	11.8	14.06	3.02	4.94	8.3
Biomass, (T. ha^−1^)	18.4	23.1	29.4	33.0	7.0	10.8	20.3
Harvest index (%)	65.5	61.1	59.1	57.1	57.0	54.2	59.0
Dry matter content of storage roots	16.5	18.2	19.1	18.1	15.5	16.7	17.4

*Rub.A* Rubona season A, *Rub.B* Rubona season B, *Kar.A* Karama season A, *Kar.B* Karama season B, *Mus.A* Musanze season A and *Mus.B* Musanze season B

With respect to SRFY and BIOM, high regression slopes (*b*_*i*_ > 1) associated with high MS deviations were observed for AC209006, EC209018, AC209023, AC209031, AC209032, AC209033, AC209034, AC209035 and EC209050 (Table 7). The *b*_*i*_ was pronounced for EC209018 with 2.42 and 2.36 for SRFY and BIOM, respectively. The accessions AC209031 and AC209033 exhibited a high values of *b*_*i*_ for total root yield, biomass yield and harvest index. The environments for which SRFY exhibited steep regression slopes (*b*_*i*_ > 1) were Rubona A, Karama A and Karama B, whereas environments for which BIOM were found to exhibit steep regression slopes (*b*_*i*_ > 1) were both seasons (A and B) at Rubona and Karama. The regression slope for HI was only pronounced (*b*_*i*_ > 1) at Musanze B.

**Table 7:** Estimates obtained using the dynamic concept of GxE interaction for storage roots yield, biomass and harvest index

	Total root yield			Biomass			Harvest Index		
	*X*_*i*_	*b*_*i*_	MS Dev. R.	*X*_*i*_	*b*_*i*_	MS Dev. R.	*X*_*i*_	*b*_*i*_	MS Dev. R.
Genotypes									
AC209003	9.83	0.98	78.42	17.41	0.83	253.15	50.28	2.97	531.68
AC209004	8.50	0.73	112.19	14.53	0.62	349.06	56.10	2.59	304.53
AC209006	12.50	1.02	75.95	21.88	1.06	180.19	59.04	−0.26	34.36
AC209007	10.00	0.81	103.40	17.70	0.66	328.89	54.79	0.80	260.61
EC209016	10.40	0.83	101.30	16.87	0.86	268.18	63.33	0.52	22.60
EC209017	11.70	1.01	76.43	19.81	0.99	213.71	59.11	1.07	69.90
EC209018	25.50	2.42	0.15	41.37	2.36	3.45	65.83	−1.61	81.35
EC209019	1070	0.59	144.59	19.08	0.65	328.02	57.26	0.61	33.94
AC209022	11.30	0.68	117.39	18.71	0.79	266.50	64.18	−0.20	14.90
AC209023	13.20	1.20	58.85	22.02	1.18	166.22	58.78	0.80	78.15
AC209024	9.40	0.70	123.00	15.56	0.69	332.66	61.52	0.71	60.79
AC209031	12.60	1.07	74.16	21.33	1.08	205.46	58.55	1.38	91.21
AC209032	13.60	1.03	73.81	22.64	1.09	159.85	62.87	−0.38	20.38
AC209033	17.10	2.04	56.04	29.12	2.09	245.68	58.45	2.24	242.01
AC209034	13.40	1.07	80.12	23.53	1.18	177.11	61.65	−0.63	34.21
AC209035	16.60	1.59	29.05	28.39	1.76	85.77	59.30	0.87	18.69
EC209036	9.00	0.80	103.96	15.56	0.70	328.97	57.07	3.24	309.91
EC209046	11.70	0.99	81.86	20.28	1.02	209.35	59.43	0.87	61.84
EC209050	12.20	1.01	81.12	20.41	1.09	201.65	62.64	0.20	11.76
EC209052	8.00	0.36	170.82	13.42	0.29	471.07	55.76	2.29	141.92
TC209054	6.50	0.49	141.14	11.31	0.37	422.08	54.00	2.27	463.95
TC209060	9.00	0.49	142.65	15.06	0.55	344.64	57.34	1.58	92.31
LSD (0.05)	0.53	0.94		0.55	0.96		2.08	-0.80	
B test			77.86**			79.49**			58.25**
Locations									
Rubona A	11.30	1.02	20.98	18.40	1.16	68.18	65.50	−0.57	63.35
Rubona B	14.00	0.97	25.87	23.10	1.03	74.68	61.10	0.22	94.76
Karama A	17.50	1.79	5.80	29.40	1.86	19.77	59.10	0.19	75.35
Karama B	19.00	1.81	12.48	33.00	1.82	30.41	57.10	0.18	31.87
Musanze A	4.00	0.19	41.06	7.00	0.05	125.74	56.90	4.05	271.77
Musanze B	5.80	0.19	40.28	10.80	0.07	127.27	54.20	1.94	64.75
LSD (0.05)	0.40	0.98		0.43	0.97		1.09	-0.63	
B test			20.56**			30.66**			5.74 ns

**Significant at the 0.01 level, Xi: mean, MS Dev. R.: MS deviations from regression, *b*_*i*_: Finlay and Wilkinson’s regression coefficient, B test: Bartlett test

**Fig. 1:**
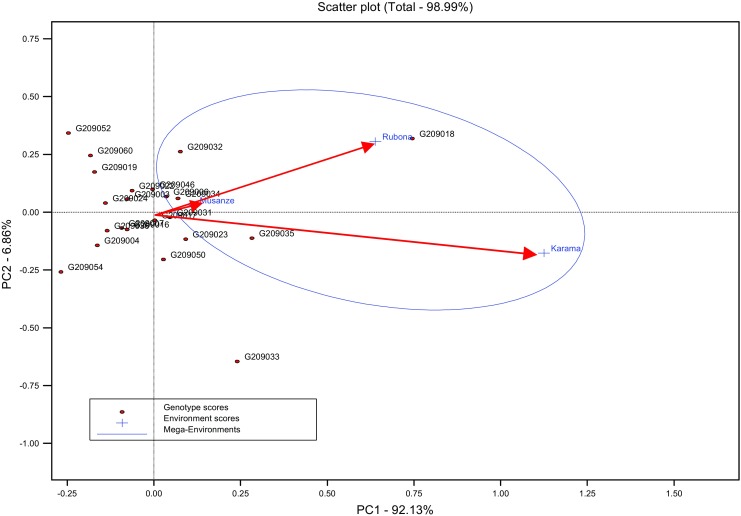
GGE bi-plot of mega-environments and environmental differences in discriminating 22 yam bean accessions for storage root yield tested in three locations during 2012A and 2012B seasons in Rwanda

The GGE bi-plot (Fig. 1) showed four high yielding environments (Rubona A, Rubona B, Karama A and Karama B) with respect to both storage roots and vines, which exhibited high positive values for the first principal component (PCA1). Low yielding environments (Musanze A and Musanze B) exhibited negative or near to zero values for PCA1. Genotypes with PCA1 scores near zero had little interaction across environments and, vice versa. Genotype and environment combinations with PCA1 scores of the same sign produced positive specific interaction effects, whereas combinations of opposite signs had negative specific interactions (for details see also Crossa et al. 1999 and Gauch 2006). Genotypes or environments on the same parallel line, relative to the ordinate, exhibited similar yields and a genotype or environment on the right side of the midpoint of this axis has higher yields than those on the left side (Fig. 1). Therefore, the medium to high-yielding genotypes (EC209018, AC209023, AC209032 C209033, AC209035) exhibited positive values to the right side for the principal component axis PCA1, with EC209018 being the overall best and also some genotypes that were found to be close to zero (AC209006, EC209017, AC209031and AC209034). Generally, EC209052, TC209054 and TC209060 were low yielding and unstable (high negative IPCA1 score), while AC209034, AC209035 and EC209046 were medium yielding and stable across environments (positive and close to zero PCA1 scores). Genotypes EC209018 and AC209033 were high yielding and very unstable across environments (high positive PCA1 score).

**Fig. 2:**
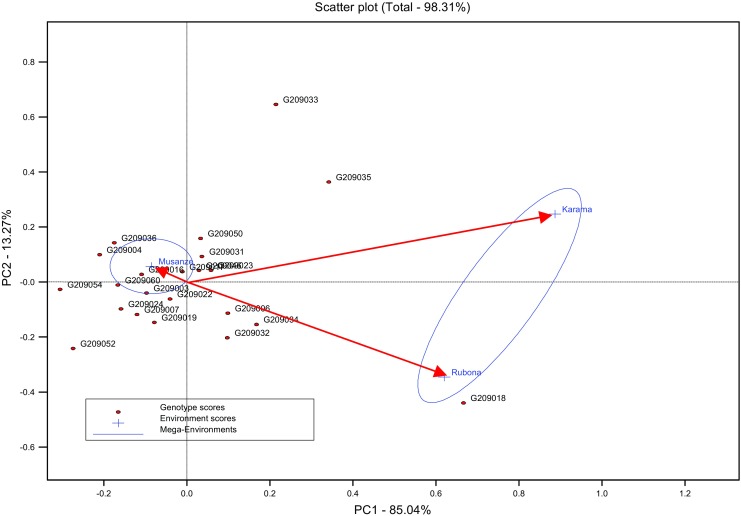
GGE bi-plot of mega-environments and environmental differences in discriminating 22 yam bean accessions for vine yield tested in three locations during 2012A and 2012B seasons in Rwanda

Low yielding environments (Musanze A and Musanze B) for both SRFY and VNY exhibited negative values with some near to zero for the first principal component axis (PCA1), whereas high-yielding environments (Karama A, Karama B, Rubona A, and Rubona B) exhibited positive or close to zero values for PCA1 (Fig. 1 and Fig. 2). Low yielding genotypes (AC209004, EC209052, TC209054 and TC209060) showed negative values for PCA1. For vine yield, the first and second principal components of the GGE analysis explained 85.04% and 13.27% of total G × E interaction sum of squares, respectively (Fig. 2). Although there was one mega environment for SRFY, most of accessions were concentrated close to the location Musanze which is low in yield potential.

## Discussions

### Effects of Genotype, Trial Sites and Seasons on Yield and Yield Components of Yam Bean

Crop varieties show wide fluctuations in their yielding abilities when grown over varied environments or agro-climatic zones (Caliskan et al. 2007; Hassanpanah 2010). The significance of genotype by environment interaction (GEI) raised the need to search for yield performance and yield stability for introduced yam bean genotypes in our study. In all environments evaluated, genotypes EC209018, AC209033 and AC209035 were found to have the highest storage root yields associated with relatively low GSL interaction effects (Table 2). The potential of every test environment across seasons showed consistency in the performance of high and low yielding locations for roots and biomass yield (Fig. 1). The consistency across seasons exhibited by the genotypes is a desirable attribute in plant breeding when genotypes perform well at sites irrespectively of environmental season conditions (Annicchiarico 2009). In this study, the cross-over interactions were observed and the GEI variance components for the introduced yam bean with respect to yield were found to be large, indicating a large diversity among accessions. The number of roots per plant did not vary among genotypes, a result similar to the findings of Zanklan et al. (2007) in West Africa. This is probably due to the fact that most cultivated yam bean species tend to produce only one storage root (Sørensen 1996). Most accessions were free from attack by insects, nematodes and diseases such as common bean mosaic virus and rust diseases on the leaves, stems or roots (results not shown). However, it might be too early to make conclusions on the susceptibility of yam beans to pest and diseases under Rwandan growing conditions since our trials were the first for the crop in Rwanda and no pest and disease pressure was established.

The magnitude of variation among locations was large which is in agreement with previous findings from yam bean trails in West Africa (Annerose 1998; Zanklan 2003; Zanklan et al. 2007). The genetic variance for storage root yield and yield components (BIOM, HI and RDM) was significant (except for number of roots), which means that considerable improvement in yield can be expected by breeding, but for number of roots, it appears that only small or limited improvement can be made (Table 4). Similar results have been reported by Zanklan (2003), where number of storage roots per plant did not vary significantly among yam bean accessions and species. In contrast to other root crops such sweetpotato and cassava (Dixon and Nukenine 1997; Grüneberg et al. 2005﻿; Sseruwu 2012), the number of yam bean roots appear to be not important as yield component.

### Heritability of Storage Root Yield and Other Related Traits

The heritability estimates (*h* ^*2*^) were relatively high for all traits, except for number of roots, vine yield and harvest index which both are highly determined by the environment. This implies that rapid selection for most of the studied traits of introduced yam bean would be possible and good genotypes can be predicted from the phenotypic attributes. Similar results have been reported for broad sense heritability estimates in sweetpotato (Martin and Jones 1986; Grüneberg et al. 2009).

Heritability estimates are fundamental for selection of the best individuals and for successful genetic improvement. This High heritability indicated that during early breeding stages, it is possible to select for storage root yield in the introduced yam beans accessions. Similar results have been reported in sweetpotato when selecting in two to three contrasting environments (Gruneberg et al. 2015). A high harvest index may be good for yam bean selection as it has large potential to increase storage yields while decreasing above ground biomass production, especially since yam bean is a seed propagated crop and does not require vegetative planting material for cultivation. However, it would be interesting to find out the effect of selection for high storage root yields on seed set in yam bean.

### Adaptability and Yield Stability of Introduced Yam Bean Accessions

The heterogeneity of GEI raised the need to select stable and high yielding genotypes. The Bartlett test in the regression model showed that genotypes EC209018, AC209033, AC209034 and AC209035 recorded high storage fresh yields compared to the population average of 11.9 t ha^−1^, and these accessions where also found to exhibit high stability based on the regression coefficient (b > 1). Similar results were observed on vine yield of the same set of four top varieties. Similar associations were found for harvest index in AC209003, 209,004, AC209031, AC209033, EC209036, EC209052 and TC209054 (Table 7). For storage root fresh yield, high regression slopes (*b*_*i*_ > 1) associated with low MS deviations were observed for accessions AC209006, EC209017, EC209018, AC209023, AC209031, AC209032, AC209033, AC209034, AC209035 and EC209050, while for biomass, high regression slopes (*b*_*i*_ > 1) were associated with low MS deviations for accessions 209,035 and 209,018. Accessions 209,006, 209,023, 209,031, 209,032, 209,033, 209,034 and 209,050 exhibited a high value of *b*_*i*_, associated with high MS deviations (Table 7) suggesting that selection of those accessions will perform better and exhibit high yield stability in high-yielding environments which are Karama and Rubona.

The GGE scatter bi-plot also demonstrated that the best yielding genotypes for SRFY also favor the environments with the highest yield which are Karama and Rubona. The best site for SRFY was Karama followed by Rubona. Low altitude zone (Karama) is suitable for major root crops, often giving high yields while Rubona (Mid altitude zone) is an average yielded environment (Ndirigue 2006; Tardif-Douglin and Rwalinda 1993). Our findings identified one mega environment for yam beans, therefore the selection fo suitable genotypes is viable since the locations used in our study represent various agro-ecological zones in Rwanda. The GGE scatter bi-plot showed the most high discriminating and suitable environments to be in Karama followed by Rubona (Fig. 2). The locations of Karama and Rubona are certainly good selecting sites for yam bean. Selection is often performed in high-yielding environments because differences between genotypes are more pronounced in high-yielding than in low-yielding environments (Annicchiarico 2002; Cooper et al. 2006). The existence of one mega-environment showed that it is not essential to have separate yam bean selection / breeding programs for various environments. Although, the high yielding locations (Rubona B, Karama A and Karama B) fell in one mega-environment, their mean performance and the mean square deviations from regression MS Dev. R (Table 7) showed that it would be efficient to evaluate yam beans in high yielding environments. These environments are the most discriminating and might offer good testing conditions for advanced as well as early testing. Genotypes selected under high yielding environments usually perform better than those selected low yielding environments when grown across a wide range of environments (Slafer and Araus 2007; Calderini and Slafer 1999). These results therefore indicated that increasing of the number of locations would not enhance the breeding and selection efficiency in yam beans under the growing conditions of Rwanda. It should be noted that Rwandan agro-ecological zones and breeding sites are stratified by altitude (ISAR 2005).

The GGE scatter plot for vine yield showed 2 M environments (Fig. 2). In case of aiming at pods or vine production for genotypes with less or no rotenone content for use as animal feed as and human consumption, a yam bean breeding program may have an advantage to screen genotypes in both mega-environments. Finally we want to note that stability of accessions’ performance in the field is also influenced by existing biotic and abiotic stresses (Cock and Hershey 1985). Resistance to biotic stresses in addition to tolerance of common abiotic factors in the environments would ensure good varietal performance. In this study, the most stable yam bean accessions (Table 7) generally had low to moderate disease and pest scores across locations (results not shown).

## Conclusion

There were large genotype-by-environment interactions associated with yield and yield components in the studied yam bean material, Nevertheless, breeding for high yielding, widely adapted yam bean accessions in Rwanda appears to be promising. The genotypes AC209034, AC209035 and EC209046 possess high adaptation and relative stability at all three locations used in our study. Accessions AC209033, AC209035 and EC209018 are recommended to be tested on-farm due to their high storage root yields and yield stability.

## Materials and Methods

### Plant Materials

Twenty two yam bean accessions, comprising of twelve *P. ahipa*, eight *P. erosus* and two *P. tuberosus* (Table 1) were introduced in Rwanda as true seed from the gene bank of International Potato Center (CIP) in Lima, Peru. These accessions were selected from *Pachyrhizus spp*. germplasm introduced in four East and Central African countries under the Ahipa project in 2010 (Heider et al. 2011). The accessions were multiplied at Rubona station in southern Rwanda to generate adequate seeds for the study. On basis of the multiplication under Rwandan growing conditions *P. ahipa* accessions were characterized as bushy plants and early maturing (4–5 months), while *P. erosus* and *P. tuberosus* accessions are climbing plants and late maturing (6 to 8 months).

### Experimental Sites

Field experiments were conducted at three sites in Rwanda, namely: Musanze, Rubona and Karama. These experimental sites represent the major agro-ecological zones of Rwanda. Musanze is located at 1850 m above sea level (m.a.s.l.) in the highland zone of Rwanda with volcanic and gravel soils that are very rich in humus and are important for potato production in Rwanda. Musanze is also characterized by highland volcanic soils, bimodal rainfall of 900 mm and temperature average of 14.5 °C, and 29°37′ East and 1°28′ South. Conversely, Rubona is located at 1650 m.a.s.l, 29°46′ East and 2.29° south and represents the mid-elevation agricultural zone of Rwanda with bimodal rainfall of 413 mm and temperature average of 19.15 °C.. The soils of the mid-elevation zone (the most important agricultural production zone in Rwanda), are granitic, light gravel loams (Tardif-Douglin and Rwalinda 1993; ISAR 2005). Then, Karama location is situated at 1350 m.a.s.l, 30°12′ East and 2°15′ South representing the low-elevation agricultural zone of Rwanda with bimodal rainfall of 345 mm and temperature of 23.5 °C. (ISAR 2005). The low-elevation zone is characterized by clay soils type rich in humus and is also suited for production of root crops such as cassava and sweetpotato (ISAR 2005; Ndirigue 2006).

### Experimental Design and Management

Across the three locations, yam beans of were planted in row-plots comprising of three ridges. The plots were replicated three times in randomized complete block design, during the two consecutive growing seasons of September 2012 and March 2013. Each row-plot was 1.8 m long and one meter apart. Seed was sown by hand per hole at 2 cm depth with a spacing of 30 cm between plants giving a planting density of 21 plants per plot. Staking was done for climbing accessions (*P. erosus* and *P. tuberosus*) to ensure upright growth and avoid ground spreading (Zanklan 2003). Reproductive pruning (usually applied in yam bean production) was done once a week across accessions and sites to increase storage root production (Zanklan et al. 2007).

### Data Collection

Data on damage due to leaf piercing insects such as aphids and mealy bugs was collected at one month after planting (MAP) and severity of rust disease especially caused by *Cercospora spp.* were scored using a hedonic scale of 1 to 5 (Zanklan 2003; Huaman 1991), where 1 represents highly resistant or no symptoms on plants, 2 represents resistant or mild symptoms on few plants, 3 mild resistant or mid symptoms on many plants, 4 susceptible with severe symptoms, and 5 very susceptible with severe symptoms. Data on these two traits was again collected just before harvesting of the crop.

Harvesting was carried out at 7 MAP by uprooting all plants in the plot and roots detached. Data was recorded on a plot basis for the number of plants harvested, total number of storage roots, weight of storage roots per plot, and weight of above ground vines (biomass), Three to five storage roots of 200 to 300 mg were collected per plot, washed clean, peeled and chopped to make a homogeneous mixture. From the mixture, two sub-samples per plot again of about 200 to 300 g were collected in paper bags and dried in an oven at 60 °C for 72 h. The dry weight of the samples was determined and the dry matter content of the storage roots was calculated using the following formula: % DM = 100 x (dry weight / fresh weight) as described by Wilken et al. (2008). Harvest index (HI) was calculated as the percentage ratio [(RDMY/Biomass) × 100]. Data on storage root fresh yield (SRFY), vine yield (VNY), fresh biomass yield (FBY = SRFY + VNY) were calculated to tha^−1^ using the area harvested.

### Data Analysis

The analysis of variance and determination of variance components for agronomic traits [number of storage roots, storage root fresh yield (SRFY) and vine yield (VNY), total biomass (BIOM), harvest index (HI) and storage root dry matter (RDM)] was performed using plant breeder statistics (PLABSTAT) computer statistical software (Utz 1997). Data were classified relative to genotype (G), location (L), season (S) and replication (R). The model statement of this analysis was *x*_*i*_ = G + L + S + GL + GS + LS + GLS + R:LS + RGLS, where G: genotype, L: location, S: season, GL: genotype by location interaction, GS: genotype by season interaction, LS: location by season, GSL: genotype by season by location interaction, R:SL: blocks within season and location and RGSL: plot error or residual of genotypes (G), seasons (S) and locations (L). Each trait *x*_*i*_ was analyzed separately for each experimental site to determine outliers, prior to combined analysis of variance following the statistical model:

$$ {Y}_{i j k l}={\upmu}_i+{g}_{i j}+{l}_{i k}+{S}_{i l}+{g l}_{i j k}+{g s}_{i j l}+{l s}_{i k l}+{g l s}_{i j k l}+ bl{(ls)}_{i n(kl)}+{\upvarepsilon}_{i j k \ln }, $$

where *g*_*ij*_, *l*_*ik*_, *S*_*il*_, *gl*_*ijk*_ *,gs*_*ijl*_, *ls*_*ikl*_ and *gls*_*ijkl*_ are the effects of genotypes, locations, seasons, genotype–location, genotypes-seasons, locations-seasons, genotypes-locations-seasons interactions, respectively, *bl*(*ls*) is the effect of blocks with locations and seasons, and other effects as given in the above statistical model.

Operative broad sense heritability (*h* ^*2*^) of observed traits was calculated with the following formula:

$$ h2=\frac{\sigma_G^2}{\left({\sigma}_G^2+\frac{\sigma_{G xS}^2}{s}+\frac{\sigma_{G xL}^2}{l}+\frac{\sigma_{G xS xL}^2}{s^{\ast } l}+\frac{\sigma_{\varepsilon}^2}{s^{\ast }{l}^{\ast } r}\right)}\times 100 $$

where σ_G_^2^, σ_G×S_^2^, σ_G×L_^2^,σ_G×S×L_^2^ and σ_ε_^2^ are the variance components due to the effect of genotype, genotype by season interaction, genotype by location interaction, genotype by season by location interaction, and plot error, respectively; and where *s,l* and *r* are the number of seasons, the number of locations and the number of plot replications, respectively. This heritability depends largely on σ_G_^2^, σ_G×S_^2^, σ_G×L_^2^,σ_G×S×L_^2^ and σ_ε_^2^ as well as the test precision determined by environments *s* and plot replications *r* (Patterson 1997).

Stability and adaptability analysis was carried out by aggregating the factor season and location into the factor environment using the ANOVA of PLABSTAT with the model statement, *x*_*i*_: E + R:E + G + GE + RGE, where E: environment, G: genotype, GE: genotype by environment interaction, RGE: plot error. This was done in combination with the PLABSTAT statement SUBINT GE to determine the stability parameters: variance of each genotype across environments, ecovalence, heterogeneity due to regression slopes and deviations from regression slopes for genotypes by environments (Gauch and Zobel 198﻿﻿8; Becker and Léon 1988; Wricke and Weber 1986). Bartlett’s test was used to test the homogeneity of variances (Gauch and Zobel 1988). Finlay and Wilkinson’s joint regression analysis (*bi*) was also used to determine adaptability and stability of genotypes across environments (Finlay and Wilkinson 1963).

The relationship between yam bean accessions and trial sites was determined with GGE bi-plot analysis. The differences due to genotype and genotype by environment interactions were investigated using the principal component analysis (PCA) of environment-centered data through GGE bi-plot analysis (Yan et al. 2000; Yan and Kang 2002). This analysis was performed with Genstat 14th edition (Payne et al. 2011). The basic model for the GGE bi-plot as described by Yan et al. (2001) as Y_ij_ = b_j_ + b_j_α_i_ + λ_l_ζ_il_η_jl_ + Ɛ_ij_,

Where Y_ij_: Average yield of i genotype in the environment j, b_j_: the average yield of all genotypes in environment j, α_i_: the main effect of genotype i, λ_n_: the singular value for principal component PC_n_, ζ_il_ and η_jl_: scores for genotype i and environment j on PC_n_, respectively, and Ɛ_ij_: the residual associated with i genotype and j environment. The high yielding clones in specific environments and mega-environments were determined through which-won-where polygon view pattern of GGE biplot GGE biplot (Yan et al. 2000; Yan and Kang 2002). The discriminating power of each environment was tested using the GGE biplots based on average environment coordination (AEC) (Yan and Kang 2002).

## Abbreviations

BIOM: Total biomass
GGE: Genotype + Genotype x Environment
GEI: Genotype by Environment Interaction
HI: Harvest Index
RDM: Root Dry Matter
SRFY: Storage Root Fresh Yield
VNY: Vine Yield
